# Our New York Letter—The Mechanical Treatment of Hernia

**Published:** 1886-04

**Authors:** Wm. Penny

**Affiliations:** New York


### OUR NEW YORK LETTER.

#### MECHANICAL TREATMENT OF HERNIA.

New York, April 1, 1886.

Editor Daniel's Medical Journal:

THE importance of the mechanical treatment of hernia was ably demonstrated in an essay read by Dr. De Yarma, before the New York Academy of Medicine, a short time since.

It was my intention to give extracts from the essay, together with a running commentary. As the essay and the discussion on it has not been published, I will have to quote from memory.

The essay dealt only with the palliative treatment, while the discussion took a much wider range.

The position taken by Dr. De Yarma that hernias were with very few exceptions benefited, and that a large per centage of hernias even of long standing can be cured, was a bold and much needed advance in surgery.

The dictum has been so long and generally accepted that hernias, except in children were practically incurable, that a defense of mechanical surgery was much needed.

The position taken by Dr. De Yarma that the inguinal canal is not clossed by adhesive inflammation, under the pressure of a truss, was a new departure from the teachings of modern surgery, and I think the time has fully arrived for this departure.

That this view is correct I think will be generally admitted by all who will carefully study the anatomy of the inguinal canal. It will be evident that the amount of pressure exerted on the walls on this canal cannot be greatly in excess of the resistance of the abdominal viscera, and even if it were, it would still remain to be proven that simple pressure produces inflammation. The pressure of a boot may produce thickening of the skin, but no pathologist would dream of calling this inflammation. Constant pressure causes atrophy by absorption; intermittent pressure causes hypertrophy from irritation. The pressure of a truss pad does produce thickening of the skin, but it would be as correct to call a bunion inflammatory as this.

Again the anatomy and physiology of the inguinal canal appear to be both lost sight of. The canal contains, besides the cord and its vessels, nothing but areola tissue. Inflammation of areola tissue is attended with the formation of pus; this is usually the case, if not always. That the formation of a phlegmon in the inguinal canal is a very rare occurance, I think that all who have had considerable surgical experience will admit. I have seen one case that occurred in the practice of a surgeon, a strong believer in the adhesive inflammation theory.

In his efforts to close the inguinal canal at the internal ring, he used a truss with a stiff spring and a conical ivory pad. This was applied over the internal ring, the object aimed at being to invaginate the external parieties and set up adhesive inflammation and close the canal at its commencement. A better instrument, if the theory was correct, could hardly have been devised; but out of some eight or ten cases that he had under treatment, inflammation occurred in only one, and that in this manner. In this patient, a sharp attack of erysipilas occurred, commencing at the point where the truss pad was applied. This resulted in the formation of abscesses along the cord. After the abscesses had run their course, and their cavities had closed, it was found that the inguinal canal was closed, and the hernia had ceased to exist. Of the remainder of the cases none were benefited and some were made worse by dilitating the internal ring. This surgeon told me subsequently that in his hospital experience while a surgeon in the English army, he had seen quite a number of cases of hernia cured with the ordinary truss, and believing in the theory of adhesive inflammation he had devised the truss which proved such a failure.

Having seen the unfortunate results of experiments to close the inguinal canal by adhesive inflammation, I was fully prepared to endorse Dr. De Yarma in utterly repudiating this theory, and I believe that the acceptance of his views will be of the greatest advantage both to the profession and to those afflicted with hernia.

The inguinal canal is only a patential cavity, which during the descent of the testicle, is patent. The testicle at that time is small and soft; after its descent the canal contracts somewhat, the cord is left to fill a part of the space, and a growth of moderately firm areola tissue flls the remaining portion of the canal. It is then no longer patent.

Infancy, as we all know, is the time of the most frequent occurrence of hernia; this is before the physiological changes that I have just enumerated, can take place; but, it is a well attested fact that any appliance that will retain the hernia in the abdominal cavity at this time will cure ninety-five per cent. of these cases. What stronger argument could be adduced in favor of the position that I have taken? The principle then underlying the mechanical treatment of hernia, consists in giving physiological rest to the canal; that is, in keeping the hernia reduced day and night until the canal has ceased to be patent.

When we come to scientifically consider the problem from this standpoint, we may reasonably hope for cures in a large number of cases that at present we consider incurable without other surgical proceedure.

Dr. De Yarmo has made another advance in the mechanical treatment of hernia by devising a method of taking the exact contour of the pelvis. This he does with a lead tape about thirty inches long and one-half inch wide and one-eighth inch thick. This he moulds to the pelvis; commencing with one end applied over the internal ring, he carries his tape across the abdomen, then over the pelvis between the crest of the illium and the trochanter major back to the center of the sacrum. This is then slipped off sideways and placed on a sheet of paper, the inner side gives the guide for a pencil mark which completes the diagram.

In double hernia the tape does not extend across the abdomen, but the end is placed over the ring of one canal and the tape is carried to the center of the sacrum. The diagram is then traced on paper and the process is reversed from the other; the two united diagrams give the shape for the spring.

The Doctor repudiates the French pattern of truss entirely. The elastic truss he considers of value as a night truss only. The English pattern, in which the spring crosses the abdomen, he prefers for single hernias. In my opinion this form of truss has but one serious objection—a tendency to produce hernia on the opposite side.

In the discussion on the essay from which I have just quoted, it was a singular oversight that heredity was not mentioned as one of the causes of hernia; nor was the size, firmness or lateness of the descent of the testicle as a cause alluded to. An abnormal length of the omentum as a cause was not mentioned, although the president suggested that stretching or elongation of the mesentery might be a factor in its production. Diarrhœas of infancy and laborious occupations of adult life were evidently from their obviousness omitted.

The Doctor's object in reading the essay, as is mine in this running commentary, is to place the treatment of hernia in the hands of the profession where it belongs; and I hope I will be pardoned for saying a few words on the subject of trusses and pads.

It has been said and repeated so often that, "there has been no changes or improvements in trusses, only slight alterations in the pads," that I wish to make here a denial of this sweeping assertion. Those making this statement are either wanting in knowledge of mechanics, or of the various appliances that have been designed for the purpose of retaining hernias. All of these have at least one object in common; that of control of the hernia, and this is about all they have in common.

It is true that a vast amount of ingenuity has been displayed in altering and improving the pads, without always improving their form or making them more effective. I shall discuss this subject after briefly noticing some of the more common forms of truss in actual use; and the mechanical principles involved in their construction.

We may divide appliances for the treatment of hernia under three heads: Ist, supports; 2nd; trusses for the retention of hernia; 3rd, trusses for the retention of hernia, and in addition, the closing of the canal permanently; so called radical cure trusses.

Reasoning a priori upon the evolution of this class of prathetic apparatus, we naturally infer that the first appliance of this kind consisted of a simple suspensory for large scrotal hernias. This was a waist belt and scrotal bag of leather or cloth. This has undergone but little change in material or form, and is still found servicable in a limited number of cases of irreducible scrotal hernia.

After the discovery of rubber, elastic webbing has in some cases been substituted for either the belt or bag, and if properly fitted, of decided advantage in selected cases. This was the oldest and simplest form of support, no effort being made to retain the hernia in the abdominal cavity. Notice the two distinct mechanical principles involved; in the one there is simple support; in the other an effort is made to reduce the hernial protrusion by elastic compression.

The next step in the evolution of this class of apparatus, we assume to be the non elastic belt with a soft pad. The surgeon had made two discoveries—one, that he could reduce a majority of hernial protrusions; the other, that he could retain the hernia by bridging the inguinal canal with his finger. In the natural process of reasoning, he arrived at the idea of substituting a mechanical device for his finger. What so simple as a leather belt with a pad. A large pad and a soft pad were essential for even moderate success. The perineal strap so obviously essential was the result of experiment.

The history of this appliance is veiled in the mists of antiquity. It was revived by an English surgeon by the name of Mackmain, in the early part of the present century; and, although mentioned in some text books on surgery, I have been informed that it has been patented in this country. The discovery of rubber made a change in this appliance by the substitution of an elastic for the non elastic belt. This truss was used in the South during the war, but has since been patented. Metal springs were used for trusses long before the discovery of rubber. Two forms of metal spring trusses have been manufactured and sold so extensively that an impression prevails that all metal spring trusses are but slight modifications of these. Of the first of these, the French pattern has the credit of being the oldest form of metal spring truss ; in this the spring acts from the same side as the hernia, and requires a large pad. It is better adapted to hiding than retaining a hernia. The second, or English, pattern is generally preferred by surgeons. This is also called the long arm truss, on account of the spring crossing the abdomen. This form of spring forms the basis of nearly all the so-called radical cure trusses in use.

To demonstrate the fact that there are other forms of truss springs in use, and involving entirely different mechanical principles, I will mention two, differing radically from these, and from each other. One of these, the White's lever truss, has a rigid bar of iron in place of pelvic spring; the spring is straight and short, the pad rotates on a pivot moving in the arc of a circle. It appears to be well adapted to those cases of hernia complicated with undescended testicle. The other is known as Ritter's radical cure truss. This has a spring crossing the anterior surface of the body, which is bent so that the convex side comes in contact with the abdominal walls. The spring carries two pads placed on short arms. A leather belt covers the spring. Tightening the belt reverses the arch of the spring, forcing the pads back upon the inquinal canals. The pressure is nearly constant. This truss has had a reputation of being very successful as a radical cure truss in adults.

It would appear from the number and variety of truss pads, that ingenuity has exhausted itself. But every once in a while some inventor brings out a new one, which, of course, solves the problem of the radical cure of hernia. There are large pads and small pads, soft pads and hard pads, pads with a universal joint, stationary pads attached, either to the end of the spring or to a short lever riveted on to it. Pads of all forms, pads to represent the end of a finger, pads to represent the ends of four fingers, plain ovoid pads, ovoid pads large at one end and small at the other, pads made to dip down into the internal ring, pads with a ridge on the face, circular pads and cup-shaped pads, rubber pads to be filled with water, or to be inflated with air, pads made of an external oval ring and an internal ovoid pad, and so on to the end of a chapter which would fill a volume. But there is one thing noticeable in looking over an assortment of trusses—that is, the preponderance of plain ovoid pads ; and we have not far to go for an explanation of this. Said a tailor: "The cutting of the shoulder seam of a coat straight, worked a revolution in the manufacture of clothing. It enabled the manufacturers to average the human body, and produce their goods on a large scale." So in the development of the hard ovoid pad—a neuclus was formed around which crystalized the manufacture of trusses on a large scale. And, while this has taken from the instrument maker, it has given nothing to the surgeon. There is one thing, however, that it fairly demonstrates ; that is, the facility with which a hernia can be retained. It is about as philosophical to use this form of pad as to wear shoes both made from the same last. When the science or art of truss fitting comes to be taught as it should be, the surgeon will both adapt the shape of the truss spring to the individual, and also adapt the pad to the space to be covered. This he can do by mapping out the size and location of the internal ring and the inguinal canal on the surface of the abdomen with a common blue pencil. Then, as the object is to retain the hernia in the abdominal cavity, he, with the knowledge that it is most easily accomplished by bridging the inguinal canal, will be able to design a suitable pad for each case.

As the text books on surgery direct a well fitting truss, but fail to say what that is, I will give a brief outline of the requisites of such a truss.

1. It shall retain the hernia in the abdominal cavity.
2. It must not give great discomfort to the patient.
3. It must remain in position after being fitted.

4. It must allow the pad to follow the respiratory movements of the abdomen.
5. It must not produce a hernia on the opposite side.
6. It must be easily fitted.

WM. PENNY, M. D.

